# The impact of paternal PTSD-like stress on cognitive behavior and hippocampal BDNF expression in adolescent male and female rat offspring: modulatory effects of lithium

**DOI:** 10.1186/s12993-026-00324-2

**Published:** 2026-03-29

**Authors:** Zahra Ebrahimkhani, Hamidreza behnoud, Ameneh-Sadat Kazemi, Batool Ghorbani Yekta

**Affiliations:** 1 Men’s Health and Reproductive Health Research Center, Shahid Beheshti University of Medical Sciences, Tehran, Iran; 2 Department of Psychology, TeMS.C. Islamic Azad University, Tehran, Iran; 3 Cognitive and Neuroscience Research Center (CNRC), TeMS.C., Islamic Azad University, Tehran, Iran, Khaqani St., Shariati Avenue, Tehran, Iran P.O. Box: 193951495,; 4 Applied Biotechnology Research Center, TeMS.C. Islamic Azad University, Tehran, Iran; 5 Herbal Pharmacology Research Center, TeMS.C. Islamic Azad University, Tehran, Iran

**Keywords:** Prenatal stress, Paternal preconception stress, Posttraumatic stress disorder (PTSD), Lithium, Brain-derived neurotrophic factor (BDNF)

## Abstract

Prenatal stress may lead to cognitive and behavioral dysfunction in the offspring. Large evidence has shown the deleterious effects of maternal stress on cognitive and behavioral functions of the offspring; however, the effect of paternal stress has not been well documented. In the present study, we aimed to investigate the effect of paternal stress (chronic electrical footshocks, post-traumatic stress disorder or PTSD-like model) on cognitive and behavioral functions, and brain-derived neurotrophic factor (BDNF) hippocampal level in both male and female offspring during adolescence. The father rat (stress-exposed) was exposed to three consecutive shocks in a fear conditioning apparatus for ten times during four weeks, in an uncertain and unpredictable schedule. Saline (0.5 mL) or lithium chloride (50 mg/kg) was intraperitoneally injected to male and female offspring during 21–41 postnatal day (PND). The results showed that paternal stress decreased locomotor activity in female offspring, and increased anxiety-like behavior in both male and female offspring, with more effect on females. Paternal stress also decreased pain subthreshold only in female offspring and impaired passive avoidance and spatial memory in both male and female offspring. Paternal stress also decreased BDNF expression level only in female offspring. However, lithium reversed most of the behavioral dysfunctions in rats’ offspring with a history of paternal stress. We concluded that paternal stress significantly impairs cognitive and behavioral function in the offspring during adolescence, with more effect on females. Also, chronic lithium treatment may reverse the deleterious effects of paternal stress.

## Introduction

Trauma is common globally, and a previous report by World Mental Health (WMH) Surveys has shown that 70% of the populations in the countries studied reported exposure to a traumatic event, ranging from 29% in Romania to 83% in Peru [1]. Trauma may lead to a wide range of psychological issues and neuropsychiatric diseases such as post-traumatic stress disorder (PTSD). PTSD is a neuropsychiatric disorder characterized by anxiety, panic-like attacks, anhedonia, and vigilance that persists for months to years after trauma experience [2]. PTSD has been considered as a ‘life sentence’ due to its relationship with elevated risk of chronic diseases, accelerated aging, and premature mortality [3, 4]. It has been estimated that PTSD affects about 3.5% of the USA population annually and about 10 percent of the USA population experience PTSD during their life [5]. Also, there seems to be a sex difference, and evidence has shown that women are diagnosed at a rate more than twice that of men, with a prevalence of 10–12% in comparison with 4–5% prevalence in men after an equivalent traumatic event [6]. In rodents, fear conditioning induced by electrical foot shock is a common method to induce a rodent model of PTSD [7, 8]. However, persistent or repetitive shock exposure may lead to aggression and has been considered as an aggression model in rodents [9, 10]. Recent studies have confirmed that repeated or intense footshock stress can provoke heightened aggression in rodents [11, 12].

PTSD and trauma experience can also affect the level and function of brain-derived neurotrophic factor (BDNF) [13, 14]. The neurotrophin BDNF is one of the most studied and well-characterized neurotrophic factors in the central nervous system (CNS) [15]. BDNF is significantly involved in synaptic plasticity, memory function, and mood regulation [16–18]. It has been shown that exposure to stress or trauma experiences drastically reduces BDNF expression in the hippocampus and the prefrontal cortex [19]. Furthermore, exposure to twenty foot-shocks with pure tones during 60 min significantly decreases dentate gyrus BDNF mRNA in rats [20]. Furthermore, early-life stress reduces BDNF level in the amygdala, leading to dendrite maldevelopment and an increased risk of mental diseases [21].

Of note, trauma experiences and stressful conditions may lead to the induction of deleterious changes in behavioral and cognitive functions of the offspring. It has been revealed that maternal social isolation severely affects learning and memory networks focused on the amygdala and the hippocampus [22]. Furthermore, maternal restraint stress during the gestational period in rats potently induces depressive-like behaviors in the offspring [23]. It has been reported that maternal stress leads to depressive-like behavior, hypothalamic–pituitary–adrenal (HPA) axis reactivity, increased pro-inflammatory cytokines, and decreased BDNF level in a strain- and sex-dependent manner in rodent offspring [24]. Moreover, prenatal maternal stress can elicit anxiety-like behaviors in the offspring [25]. On the contrary, the potential deleterious effect of paternal stress on cognition and behavior has not been well studied in the offspring. It has been demonstrated that chronic social defeat stress exposed to male mice induces depressive- and anxiety-like behaviors in both male and female offspring [26]. In addition, paternal stress is significantly related to children’s emotional problems at 2 years [27]. However, there is limited evidence on the effects of paternal stress (before mating) on offspring cognition and behavior.

Lithium has been considered as the most effective medication in psychiatry because it has disease-modifying, not just symptomatic, effects [28]. Lithium is effective not only for bipolar disorder but also for prevention of suicide, episodes of unipolar depression and mood temperaments [28]. Also, lithium may be effective in the management or alleviation of PTSD symptoms. A past study has shown the efficacy of lithium for irritability in individuals with PTSD [29]. In a previous report, James Wallace suggested that the administration of lithium for a brief interval to traumatized individuals at risk for PTSD within the time period after trauma and before the onset of symptoms may potently forestall the development of PTSD via impairment of long-term potentiation (LTP) [30]. It has been reported that lithium carbonate reduces the risk of acute suicidality in individuals with PTSD [31]. In contrast, it has been shown that comorbidity of PTSD with bipolar disorder reduces the efficacy of lithium treatment [32]. Lithium also affects the expression level of BDNF. Previous research has shown that chronic lithium treatment elevates the expression level of BDNF in the rat brain [33]. Chronic and low-dose lithium treatment significantly upregulates BDNF production in primary neuronal cell culture [34]. On the contrary, it has been shown that lithium-induced changes in BDNF level may vary depending on the duration of lithium exposure and particular brain regions exposed to lithium [35]. Also, a previous study has shown no significant changes in BDNF mRNA and extracellular level following lithium treatment in rats [36].

According to the mentioned findings, we aim to investigate the effect of paternal PTSD and lithium treatment on mood state and BDNF hippocampal expression level in male and female rat offspring.

Despite extensive evidence on the adverse effects of maternal prenatal stress, remarkably little is known about the transgenerational impact of paternal stress exposure prior to conception, particularly with respect to sex-specific neurobehavioral outcomes in offspring. Moreover, the potential of lithium as an early-life intervention to mitigate paternal stress–induced impairments in offspring cognition, behavior, and neuroplasticity markers such as BDNF remains poorly understood. Therefore, the present study aimed to investigate whether paternal PTSD-like stress alters behavioral performance, cognitive functions, and hippocampal BDNF expression in adolescent male and female rat offspring, and to examine whether postnatal lithium treatment can reverse these alterations [27, 37].

## Materials and methods

### Animals

In our study, we utilized 56 Wistar rats (28 males and 28 females), all were 6 weeks old and weighing between 140 and 160 g, along with two adult male rats (the control father and the stress-exposed father). Six-week-old rats were selected because this age corresponds to late adolescence in rodents, a developmental period characterized by heightened neuroplasticity and sensitivity to environmental stressors, making it particularly suitable for assessing stress-related behavioral and cognitive outcomes [38]. These rats were accommodated in Plexiglas cages, with four rats per cage. The environment was maintained on a 12-h light/dark cycle, with lights turned on at 7:00 a.m., and a consistent temperature of 22 ± 1 °C. Each of our experimental subsets comprised six rats of each sex. These rats were all bred and raised at the Cognitive Neuroscience Lab, located in the Medicinal Plants Research Center, Institute of Medicinal Plants, ACECR, Karaj, Iran. They had unrestricted access to both food and water. All experimental procedures were carried out during the daylight hours, specifically between 9:00 a.m. and 3:00 p.m. We ensured that our experimental approach adhered to the guidelines set by the National Institutes of Health for the Care and Use of Laboratory Animals [39]. We also adhered to the ARRIVE guidelines in designing and reporting this experiment. Experimental timeline and behavioral test order. Offspring were weaned at postnatal day (PND) 21. From PND21 to PND41, offspring received daily i.p. injections of saline (0.5 mL) or lithium chloride (50 mg/kg) as specified. Behavioral testing was conducted in the following order to minimize test interference: open field (PND35), hot plate (PND36), marble burying (PND37), passive avoidance training (PND38) and testing (PND39), Morris water maze training (PND39–PND40) and probe test (PND41). Twenty-four hours after the last behavioral test (PND42), animals were deeply anesthetized and tissues were collected [38].

### Fear conditioning apparatus (Induction of chronic electrical footshocks)

The fear conditioning apparatus was a transparent Plexiglas chamber with metal rods on its bottom which were connected to a shock device, and a speaker was placed on the top of the chamber (Tajhiz-Gostar Omid Iranian Co, Tehran, Iran). The strength and duration of the shock, and the frequency and duration of the sound, were adjustable [40, 41]. On the day of shock induction, the father rat was given three shocks of 0.8 mA for 3 s. Three seconds before each shock, a sound with a volume of 75 dB and a duration of 3 s was emitted. There was an interval of thirty seconds between each shock. After three shocks, the rat was removed from the apparatus. This process was repeated ten times over the next four weeks, in an uncertain and unpredictable schedule. After this period, the father rat was transferred to a cage with six female rats. As mentioned before, fear conditioning induced by electrical foot shock is a common method to induce a rodent model of PTSD [7, 8]. However, persistent or repetitive shock exposure may lead to aggression and has been considered to induce an aggression model in rodents [9, 10]. Recent studies have confirmed that repeated or intense footshock stress can provoke heightened aggression in rodents [11, 12]. In the present research, we used persistent and repetitive shock exposure during four weeks, leading to the induction of a PTSD-like rat model and an aggression model. The father rat of the present study showed complete freezing in the apparatus and was aggressive in its cage. Breeding schedule and paternal exposure timeline. The paternal stress protocol was conducted prior to mating. Immediately after completing the 4-week footshock regimen, the stressed male was paired with six nulliparous females for 48 h (two dark/light cycles) for timed mating. The control father underwent identical handling and apparatus exposure without electrical shock (sham) on the same schedule and was also paired with six nulliparous females for 48 h. Males were removed immediately after the mating period and housed separately; they had no contact with dams during gestation or with pups during the pre-weaning phase [42, 43].

### Open field test

Locomotor activity was assessed through the open field test (OFT) (Tajhiz-Gostar Omid Iranian Co, Tehran, Iran). The apparatus was a transparent Perspex box measuring 30 × 30 × 40 cm, divided into 16 uniform squares. Locomotor activity was quantified by counting the number of squares traversed within a 300-s session [44]. Furthermore, we evaluated anxiety-like behavior by recording the time the rats spent in the central four squares of the field. A tendency to remain near the field’s walls was interpreted as an indication of anxiety [45]. All behavioral tests were scored by observers blinded to the animals’ group assignments to minimize observer bias.

### Hot plate

To evaluate pain subthreshold, we employed a hot plate (HP) apparatus (Tajhiz-Gostar Omid Iranian Co, Tehran, Iran). This device consists of a plate that is heated by an electric current. Before each test, the HP plate was cleaned with 70% ethanol. Rats were then placed on the heated plate, and a timer was started. The moment the rats began to lick their paws or change their walking pattern, the pain subthreshold latency was recorded. The apparatus was maintained at 50 °C, with a maximum cut-off time of 100 s to ensure the safety of the animals [46].

### Marble burying test

The marble burying test is a widely used technique to evaluate obsessive–compulsive disorder (OCD)-like behaviors in rodents. In this test, standard glass toy marbles (varied styles/colors, ~ 15 mm diameter, 5–6 g each) were gently placed on top of the bedding in the rat’s home cage. They were arranged in two rows of five (total ten marbles). It’s important to note that the number of marbles used can vary (typically 4 to 25) depending on the arena layout [47]. To assess OCD-like behavior, we recorded the number of marbles buried by the rodents over a 30-min period.

### Forced swim test

To assess depressive-like behavior, the forced swim test (FST) was conducted. Each rat was placed individually in a transparent cylindrical tank (50 cm height, 20 cm diameter) filled with water at 25 ± 1 °C to a depth of 30 cm. The rat remained in the water for 6 min; the first 2 min were for acclimation, and immobility (cessation of active swimming except for movements necessary to keep the head above water) was recorded during the last 4 min. All rats were dried and warmed immediately after the test and then returned to their home cages.

### Shuttle box

The shuttle box apparatus is divided into two equal compartments (25 × 25 × 25 cm). One compartment is illuminated (light compartment) while the other remains dark. Both compartments have a grid floor and Plexiglas walls, separated by a guillotine door. During training, each rat was placed in the light compartment and allowed to acclimate for 60 s. When the guillotine door was opened and the rat entered the dark compartment, the door was closed and a 2-s foot shock (0.5 mA, 50 Hz) was delivered through the grid floor. After a 20-s interval post-shock, the rat was returned to its cage.

Twenty-four hours after training, the test session was conducted. The rat was reintroduced to the light compartment, and the latency before it entered the dark compartment was recorded, up to a maximum of 300 s, as a measure of memory retention [48].

### Morris water maze

The Morris water maze (MWM) is a well-established tool for assessing spatial learning and memory in rodents [49]. (The duplicate reference to the MWM as well-established has been removed.) The MWM setup consists of a circular tank divided into four quadrants and filled with water (circular pool with a diameter of 150 cm and a wall height of 60 cm). The pool was filled with water to a depth of 30 cm, and the water temperature was maintained at 22 ± 1 °C throughout all trials. The procedure is split into two phases: a training session (memory acquisition) and a probe test session (memory retrieval). The training session encompasses eight trials, each starting from one of four distinct positions (north, south, west, and east) evenly spaced around the maze’s perimeter. For each trial, a rat was placed at the corner of a quadrant. A hidden platform (circular platform with a diameter of 10 cm) was submerged 2 cm below the water’s surface in the target quadrant (north-west), making it invisible to the rat. Each rat had 60 s to find the platform. Upon finding it, the rat stayed on the platform for 20 s, allowing it to internalize spatial cues and their relative positions on the surrounding tank walls. Subsequently, the rat was returned to a holding cage for a 20-s break before the next trial. If a rat failed to locate the platform within 60 s, the experimenter guided it to the platform, where it remained for 20 s. For data analysis, the eight training trials were grouped into two blocks (section 1: trials 1–4, section 2: trials 5–8) to evaluate learning progress over the session. Metrics recorded during training included time spent in the target quadrant, distance swum in that quadrant, and swimming speed (as an indicator of motor function). Behavioral tracking and data acquisition were performed using a video-based tracking system connected to a digital camera mounted above the maze and analyzed using EthoVision XT software (Version 14, Noldus Information Technology, Wageningen, The Netherlands) [50]. Although brief, this 20-s inter-trial interval was applied uniformly to all groups to maintain training intensity while limiting any impact on memory consolidation across trials. The probe test session was conducted 24 h post-training: each rat was given a 60-s free swim with the hidden platform removed (probe test). A longer duration and greater distance in the target quadrant were indicative of better memory performance. The non-spatial visible platform test was then conducted after the probe trial: the platform was raised 2 cm above the water and marked with aluminum foil at the center of the north-east quadrant. This test evaluated potential non-specific factors such as motor, visual, or motivational capabilities, unrelated to spatial learning [51, 52]. We did not include a visible platform trial prior to hidden-platform training in order to avoid influencing the rats’ initial spatial learning; instead, the visible platform test was performed after the probe trial to confirm that any performance deficits were not due to visual or motor impairments.

### Anesthesia and euthanasia

Anesthesia and euthanasia. Prior to tissue collection, rats were anesthetized with ketamine (90 mg/kg, i.p.) and xylazine (10 mg/kg, i.p.). Once surgical anesthesia was confirmed (absence of righting and paw-withdrawal reflexes), euthanasia was performed by decapitation, in accordance with the AVMA Guidelines for the Euthanasia of Animals (2020) and our institutional approvals. [53]

### Real-time PCR

*Total RNA extraction and cDNA preparation:* From 100 mg of hippocampal tissue, total RNA was isolated using the Qiazol lysis reagent (Qiazol, USA) and collected in a sterilized, RNase-free container. The NanoDrop ND-100 spectrophotometer (Thermo Scientific, Waltham, MA, USA) was used to determine RNA concentration and purity by measuring absorbance at 260 nm and 280 nm (A260/A280). Following the manufacturer’s guidelines, RNA was transcribed into complementary DNA (cDNA) using the DNase I first-strand synthesis system for RT-PCR (Fermentas, Germany).

Real-time PCR assays were performed using the Takara SYBR Premix Ex Taq II (Tli RNaseH Plus, 2X) on a StepOnePlus Real-Time PCR System (Applied Biosystems) in a final volume of 20 μL. For each reaction, 2 μL of cDNA was added. Primer pairs were optimized to an annealing temperature of 64 °C. The standard curve method was employed for quantification of the target gene. Each sample was run in duplicate, and average values were used for analysis. Authenticity of PCR outcomes was confirmed by the presence of a single melting curve peak. To verify amplicon length, PCR products were also visualized on a 2.5% agarose gel [54].

*Oligonucleotide set design:* GAPDH served as the reference gene to normalize target gene expression. Primers were specifically designed to amplify BDNF. (See Table 1).

**Table 1:** Primer sequences for BDNF and GAPDH genes for polymerase chain reaction (PCR)

Gene	Forward primer	Reverse primer
BDNF	TGCAGGGGCATAGACAAAAGG	CTTATGAATCGCCAGCCAATTCTC
GAPDH	TGACATCAAGAAGGTGGTGAA	CCCTGTTGCTGTAGGCGTATT

### Experimental groups

This study consisted of 8 groups (n = 7 per group):

- Group 1: Male offspring of control fathers, receiving saline (0.5 mL, i.p.) from PND21 to PND41.
- Group 2: Female offspring of control fathers, receiving saline (0.5 mL, i.p.) from PND21 to PND41.
- Group 3: Male offspring of control fathers, receiving lithium (50 mg/kg, i.p.) from PND21 to PND41.
- Group 4: Female offspring of control fathers, receiving lithium (50 mg/kg, i.p.) from PND21 to PND41.
- Group 5: Male offspring of stress-exposed fathers, receiving saline (0.5 mL, i.p.) from PND21 to PND41.
- Group 6: Female offspring of stress-exposed fathers, receiving saline (0.5 mL, i.p.) from PND21 to PND41.
- Group 7: Male offspring of stress-exposed fathers, receiving lithium (50 mg/kg, i.p.) from PND21 to PND41.
- Group 8: Female offspring of stress-exposed fathers, receiving lithium (50 mg/kg, i.p.) from PND21 to PND41.

### Statistical analyses

Data analysis was conducted using SPSS software (Version 26). Two-way ANOVA was used for most comparisons, followed by post hoc Tukey tests when appropriate. For Morris water maze training data, a three-way repeated-measures ANOVA was applied, with trial block as the within-subject factor and paternal stress and lithium treatment as between-subject factors. The results are presented as mean ± S.D. A P-value < 0.05 was considered statistically significant.

## Results

### OFT

**Fig. 1:**
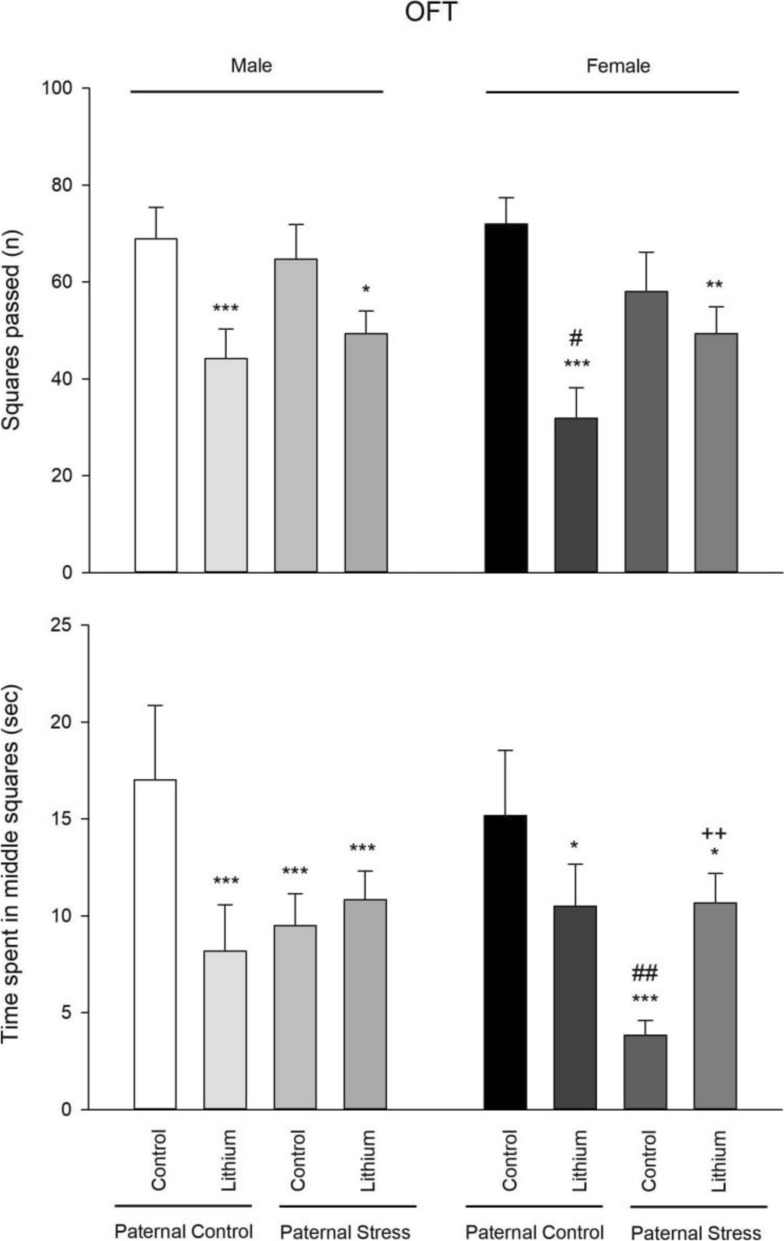
Outcomes from the open field test are presented, with the upper section detailing locomotor activity and the lower section focusing on anxiety-like behavior across all test groups (n = 6). During the period from 21 to 41 PND, lithium was administered intraperitoneally at a concentration of 50 mg/kg. Meanwhile, the control rats were given a saline solution. Notably, significant differences are indicated: ***P < 0.001 and **P < 0.01 and *P < 0.05 compared to its’ related control group of paternal control, and ##P < 0.01 and #P < 0.05 when compared to respective male group

Locomotor activity- The results of one-way ANOVA showed that there was a significant difference between male groups (F3, 23 = 14.65, P = 0.000). Post hoc Tukey test also showed that lithium decreased locomotor activity in paternal control rats (P = 0.000) and in paternal PTSD rats (P = 0.009) in comparison with control rats. The results of one-way ANOVA showed that there was a significant difference between female groups (F3, 23 = 26.80, P = 0.000). Post hoc Tukey test also showed that locomotor activity was decreased in all groups (control and lithium + paternal PTSD rats: P = 0.000; paternal PTSD rats: P = 0.002) in comparison with control rats. There were no differences between males and females (Fig. 1).

Anxiety- The results of one-way ANOVA showed that there was a significant difference between male groups (F3, 23 = 14.39, P = 0.000). Post hoc Tukey test also showed that anxiety-like behavior was increased in all experimental groups in comparison with control rats (paternal control + lithium: P = 0.000, paternal PTSD: P = 0.000, and paternal PTSD + lithium: P = 0.002). The results of one-way ANOVA showed that there was a significant difference between female groups (F3, 23 = 27.69, P = 0.000). Post hoc Tukey test also showed that anxiety-like behavior was increased in all groups (paternal PTSD: P = 0.000, paternal control + lithium: P = 0.007, and paternal PTSD + lithium: P = 0.002) in comparison with control rats. Furthermore, independent t-test showed that female paternal PTSD rats had more anxiety-like behavior than males (P = 0.002). There were no other differences between males and females (Fig. 1).

### Pain threshold

**Fig. 2:**
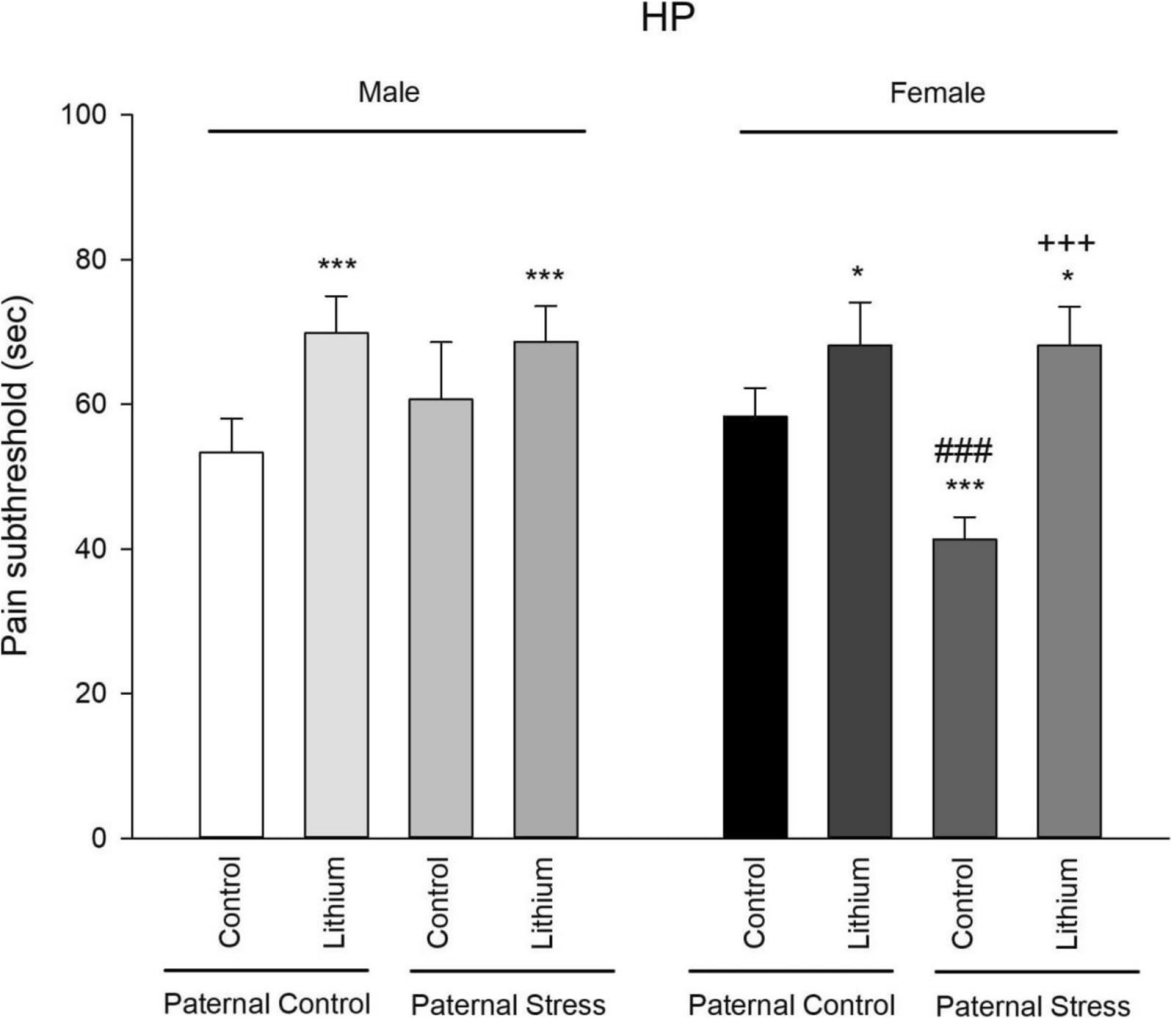
Displayed are the outcomes from the hot plate test, measuring pain subthreshold across all groups (n = 6). From 21 to 41 PND, rats were administered lithium intraperitoneally at a concentration of 50 mg/kg. For controls, a saline solution was given during the same period. Significant markers are as follows: ***P < 0.001 and **P < 0.01 compared to its’ related control group of paternal control; ###P < 0.001 when compared to respective male group; and +++P < 0.001 when compared to the control paternal stress group

The results of one-way ANOVA showed that there was a significant difference between male groups (F3, 23 = 10.56, P = 0.000). Post hoc Tukey test also showed that lithium increased pain subthreshold in paternal control rats (P = 0.000) and in paternal PTSD rats (P = 0.009) in comparison with control rats. The results of one-way ANOVA showed that there was a significant difference between female groups (F3, 23 = 43.27, P = 0.000). Post hoc Tukey test also showed that lithium increased pain subthreshold in paternal control and paternal PTSD rats (P = 0.009) in comparison with control rats. Also, pain subthreshold was decreased in paternal PTSD rats (P = 0.000) in comparison with control rats. Lithium reversed decreased pain subthreshold in paternal PTSD rats. Furthermore, independent t-test showed that female paternal PTSD rats showed a decreased pain subthreshold than males (P = 0.000). There were no other differences between males and females (Fig. 2).

### MBT

**Fig. 3:**
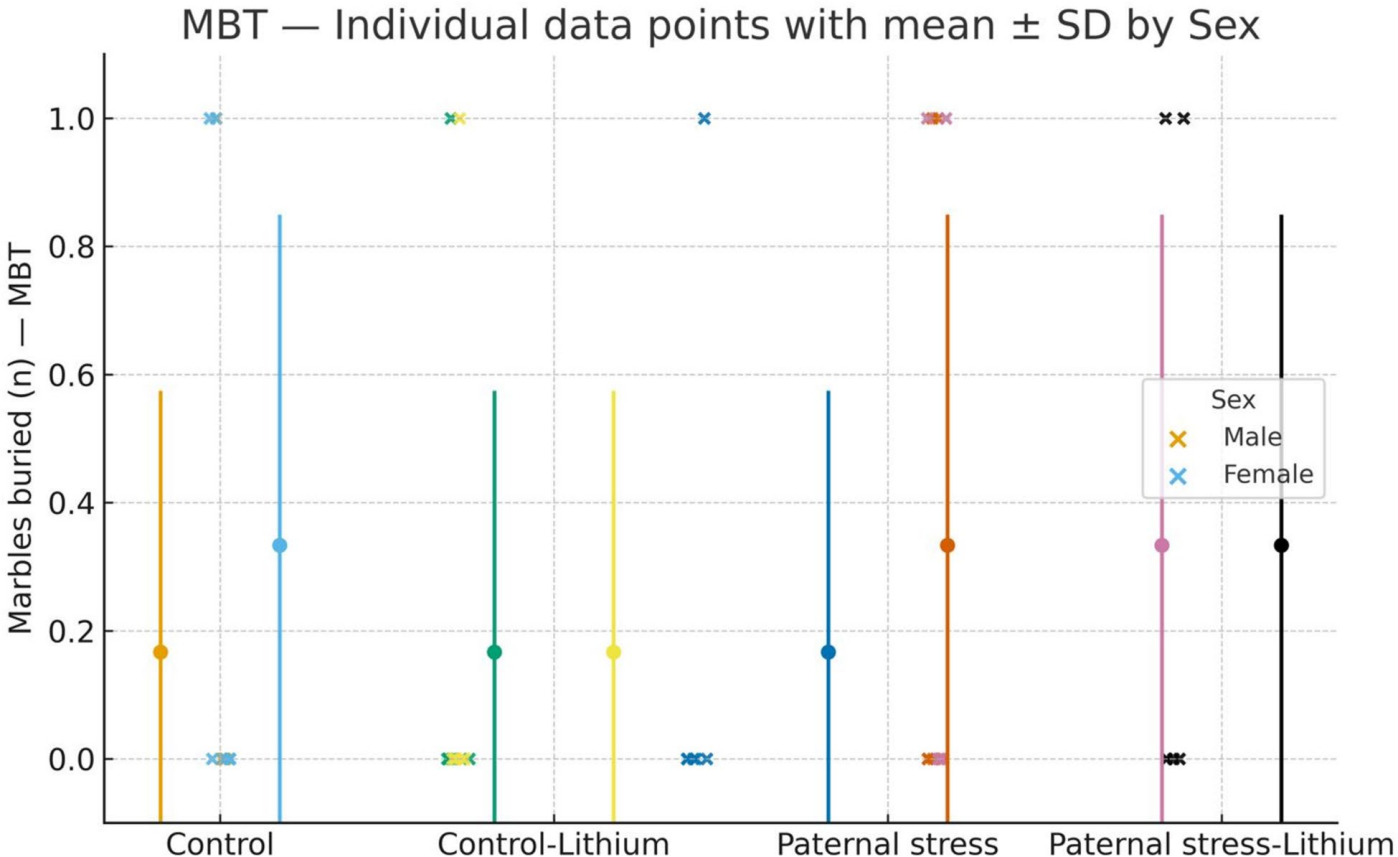
Marble Burying Test (MBT) shown as individual data points. This illustrates the findings from the marble burying test, which evaluates OCD-like behaviors in all groups (n = 6). Lithium, at a dose of 50 mg/kg, was given intraperitoneally from 21–41 PND, while control rats were administered saline during the same timeframe

The results of one-way ANOVA showed that there was not a significant difference between male groups (F3, 23 = 0.22, P = 0.883). The results of one-way ANOVA showed that there was not a significant difference between female groups (F3, 23 = 0.17, P = 0.914). Also, there were no differences between males and females (Fig. 3).

### FST

**Fig. 4:**
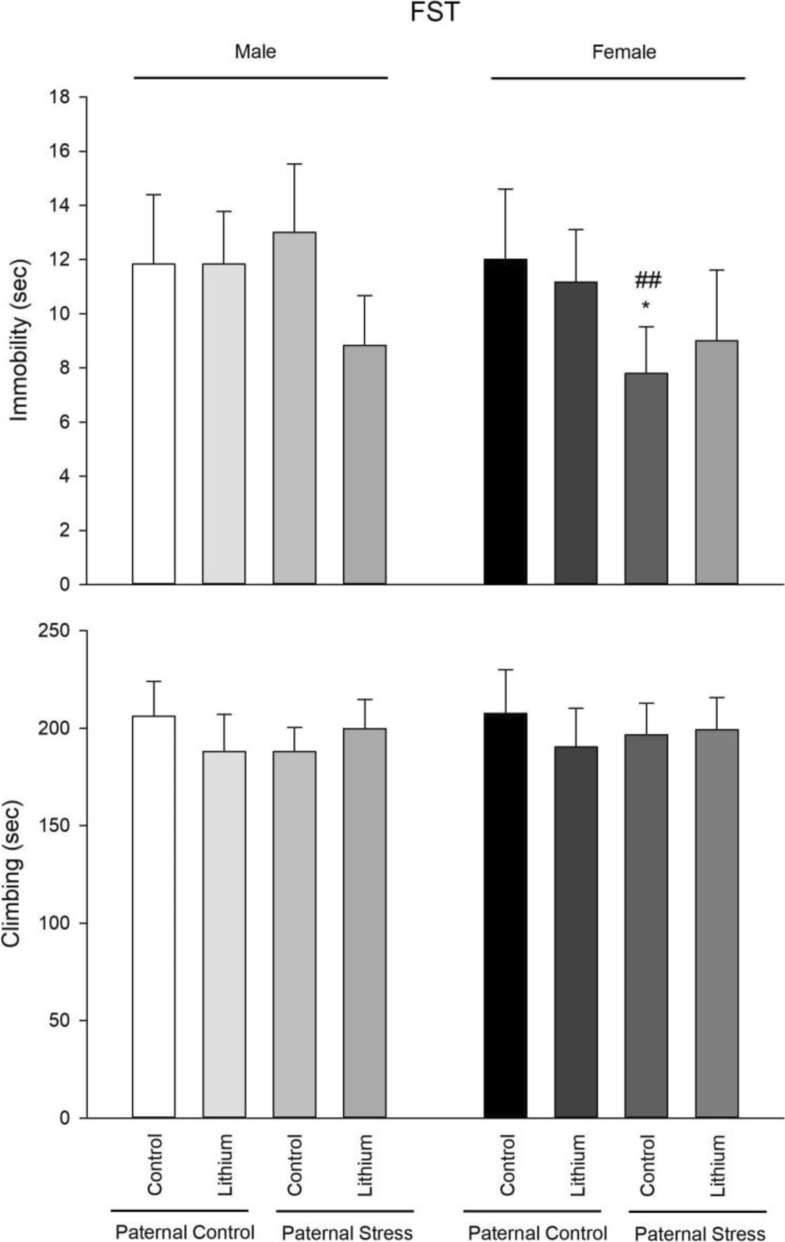
Presented here are the results from the forced swim test, which gauges depressive-like behaviors across all tested groups (n = 6). From 21–41 PND, rats received an intraperitoneal injection of lithium at 50 mg/kg. Control rats, on the other hand, were given a saline solution. Noteworthy statistical differences include: *P < 0.05 compared to its’ related control group of paternal control, and ##P < 0.01 when compared to respective male group

Immobility- The results of one-way ANOVA showed that there was a significant difference between male groups (F3, 23 = 3.79, P = 0.027). Post hoc Tukey test also showed that lithium only decreased immobility in paternal PTSD rats in comparison with paternal control rats (P = 0.021). The results of one-way ANOVA showed that there was a significant difference between female groups (F3, 23 = 5.22, P = 0.008). Post hoc Tukey test also showed that immobility was decreased in female with paternal PTSD (P = 0.023). Furthermore, independent t-test showed that female paternal PTSD rats showed a decreased immobility than males (P = 0.006). There were no other differences between males and females (Fig. 4).

Climbing- The results of one-way ANOVA showed that there was not a significant difference between male groups (F3, 23 = 1.83, P = 0.173). The results of one-way ANOVA showed that there was not a significant difference between female groups (F3, 23 = 0.86, P = 0.476). Also, there were no differences between males and females (Fig. 4).

### Passive avoidance memory

**Fig. 5:**
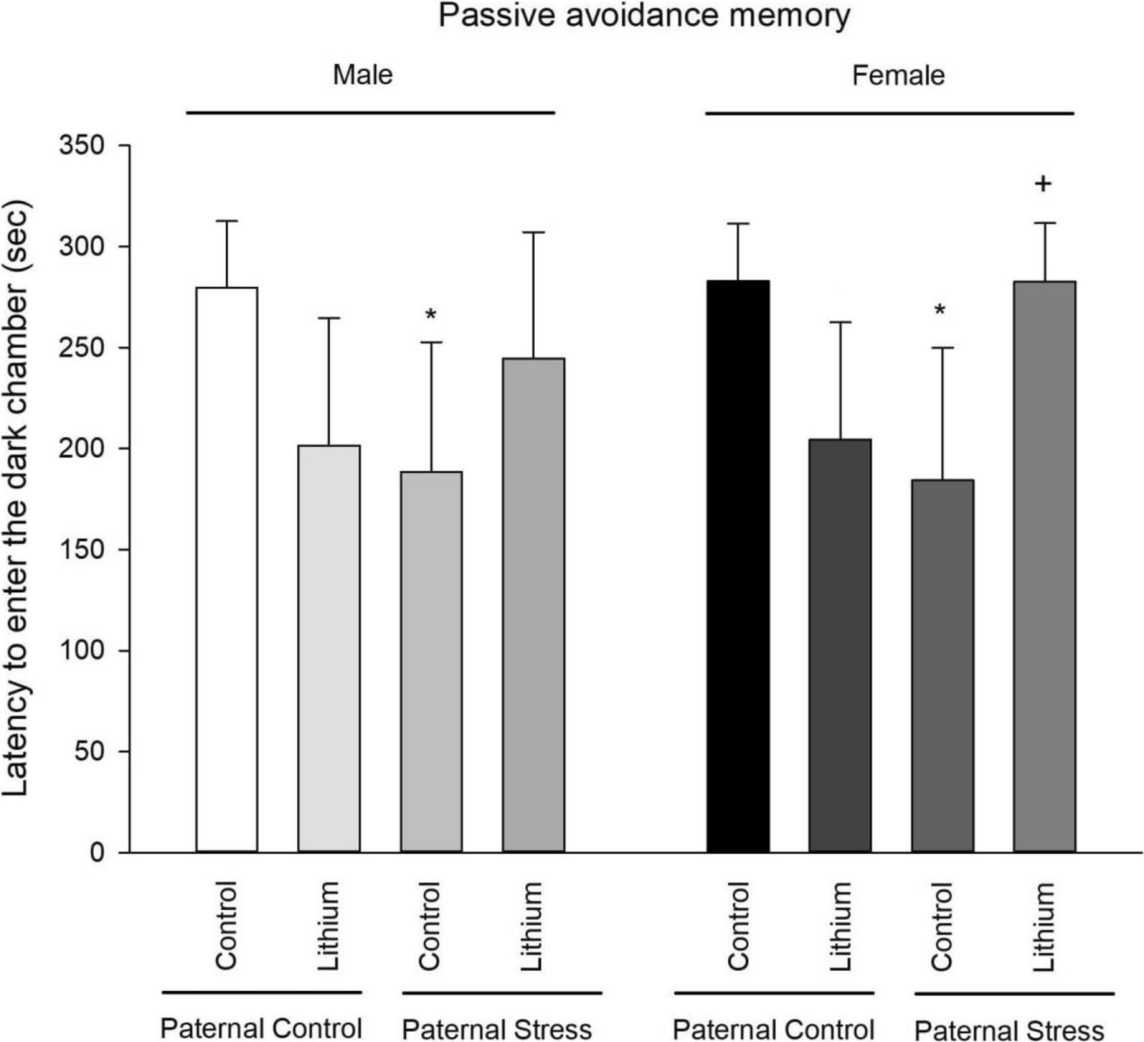
This figure showcases the outcomes from the shuttle box test, assessing passive avoidance memory across all groups (n = 6). Rats were administered lithium intraperitoneally at a concentration of 50 mg/kg from 21 to 41 PND. In contrast, control rats were given saline during this period. Notable statistical markers 29include: *P < 0.05 when compared to its’ related control group of paternal control; +P < 0.05 when compared to respective male group

The results of one-way ANOVA showed that there was a significant difference between male groups (F3, 23 = 3.46, P = 0.036). Post hoc Tukey test also showed that passive avoidance memory was impaired in paternal PTSD rats (P = 0.040) in comparison with control rats. While, lithium reversed passive avoidance memory impairment in paternal PTSD rats. The results of one-way ANOVA showed that there was a significant difference between female groups (F3, 23 = 6.92, P = 0.002). Post hoc Tukey test also showed that lithium impaired passive avoidance memory in paternal control rats (P = 0.047) and passive avoidance memory was impaired in paternal PTSD rats (P = 0.048) in comparison with control rats. While, lithium reversed passive avoidance memory impairment in paternal PTSD rats. Also, there were no differences between males and females (Fig. 5).

### Spatial learning

The results of one-way ANOVA showed that there was a significant difference between male groups for escape latency 1 (F3,20 = 11.59, P = 0.000), escape latency 2 (F3,20 = 22.57, P = 0.000), traveled distance 1 (F3,20 = 12.15, P = 0.000), traveled distance 2 (F3,20 = 19.54, P = 0.000), but not for swimming speed 1 (F3,20 = 1.31, P = 0.986) and swimming speed 2 (F3,20 = 0.95, P = 0.058). Post hoc Tukey test also showed that paternal PTSD-like model increased escape latency 1 & 2 (P = 0.000) and traveled distance 1 & 2 (P = 0.000) in comparison with controls (control offspring of control father). The results of one-way ANOVA showed that there was a significant difference between female groups for escape latency 1 (F3,20 = 12.44, P = 0.000), escape latency 2 (F3,20 = 23.51, P = 0.000), traveled distance 1 (F3,20 = 15.65, P = 0.000), traveled distance 2 (F3,20 = 24.48, P = 0.000), but not for swimming speed 1 (F3,20 = 2.01, P = 0.113) and swimming speed 2 (F3,20 = 0.39, P = 0.755). Post hoc Tukey test also showed that paternal PTSD-like model increased escape latency 1 & 2 (P = 0.000) and traveled distance 1 & 2 (P = 0.000) in comparison with controls (control offspring of control father).

**Fig. 6:**
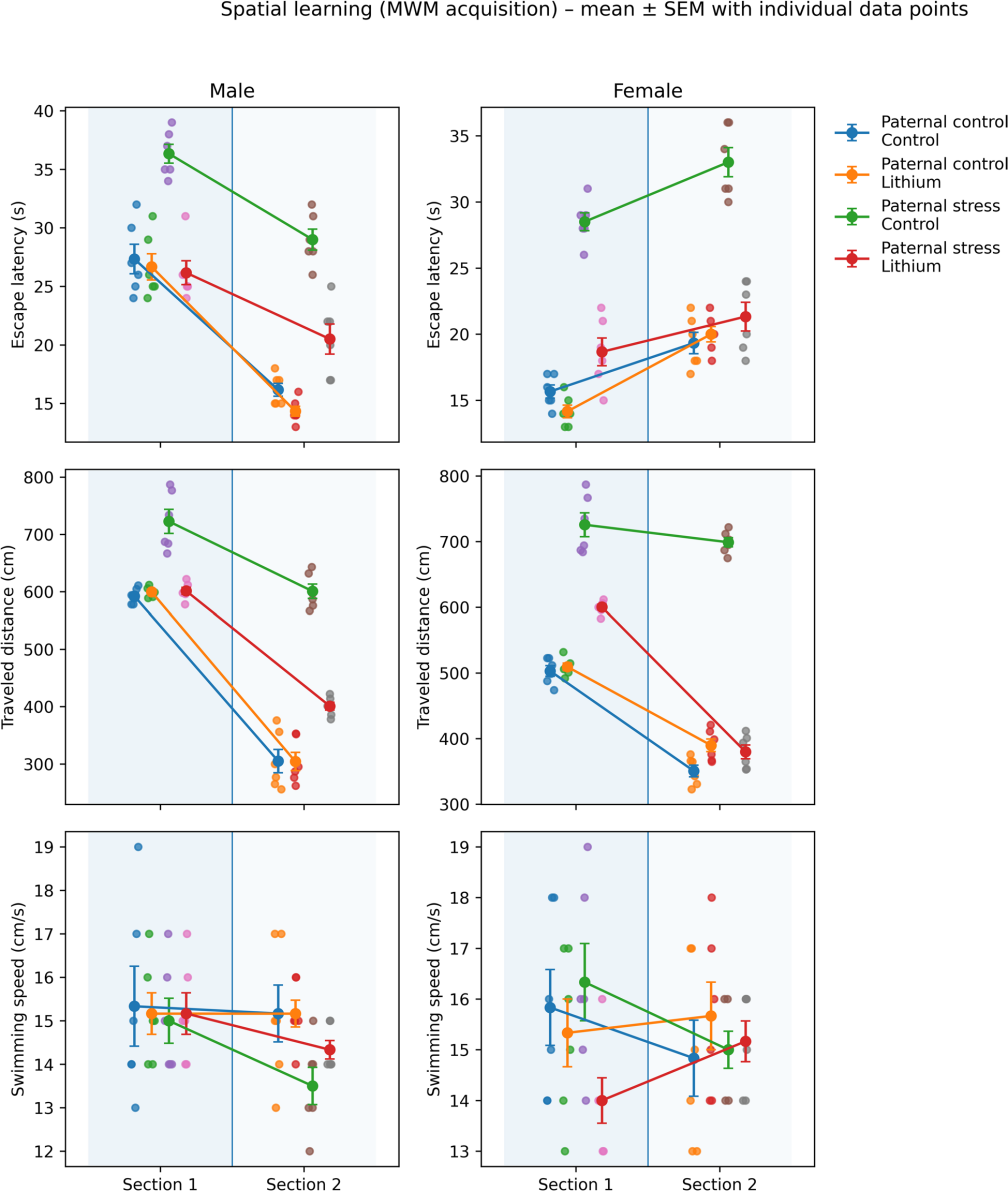
Morris water maze performance assessing spatial learning during the training (acquisition) phase in all experimental groups (n = 6 per group). Rats received intraperitoneal injections of lithium (50 mg/kg) from postnatal day (PND) 21 to 41, while control animals received saline over the same period. Training consisted of eight trials grouped into two sections (section 1: trials 1–4; section 2: trials 5–8). Data are presented as mean ± SEM and displayed as line graphs to illustrate learning progression across training sections

The results of three-way ANOVA for escape latency revealed that the effect of paternal PTSD-like model (F1.40 = 80.58, P = 0.000) and section (F1.40 = 232.69, P = 0.000) was significant, while the effect of sex (F1.40 = 0.71, P = 0.532), paternal PTSD-like model*sex (F1.40 = 1.01, P = 0.453), paternal PTSD-like model*section (F1.40 = 2.23, P = 0.214), sex*section (F1.40 = 1.81, P = 0.192), and paternal PTSD-like model*sex* section (F1.40 = 1.40, P = 0.153) was not significant. Post hoc Tukey test revealed that all groups (except male and female offspring of paternal PTSD-like model) showed decreased time spent in section 2 (P = 0.000). However, male and female offspring of paternal PTSD-like model did not show changes in time spent in section 2. The results of three-way ANOVA for traveled distance revealed that the effect of paternal PTSD-like model (F1.40 = 84.44, P = 0.000) and section (F1.40 = 262.17, P = 0.000) was significant, while the effect of sex (F1.40 = 0.55, P = 0.650), paternal PTSD-like model*sex (F1.40 = 0.91, P = 0.531), paternal PTSD-like model*section (F1.40 = 1.15, P = 0.466), sex*section (F1.40 = 1.41, P = 0.493), and paternal PTSD-like model*sex* section (F1.40 = 1.97, P = 0.523) was not significant. Post hoc Tukey test revealed that all groups (except male and female offspring of paternal PTSD-like model) showed decreased traveled distance in section 2 (P = 0.000). However, male and female offspring of paternal PTSD-like model did not show changes in traveled distance in section 2 (Fig. 6).

### Spatial memory

Spatial memory (time spent)—The results of one-way ANOVA showed that there was a significant difference between male groups (F3, 23 = 8.22, P = 0.000). Post hoc Tukey test also showed that lithium decreased time spent in the target quadrant in control rats (P = 0.000) in comparison with control rats. Also, time spent in the target quadrant was decreased in paternal PTSD rats (P = 0.033), while lithium partly reversed it, although it was not significant. The results of one-way ANOVA showed that there was a significant difference between female groups (F3, 23 = 18.71, P = 0.000).

**Fig. 7:**
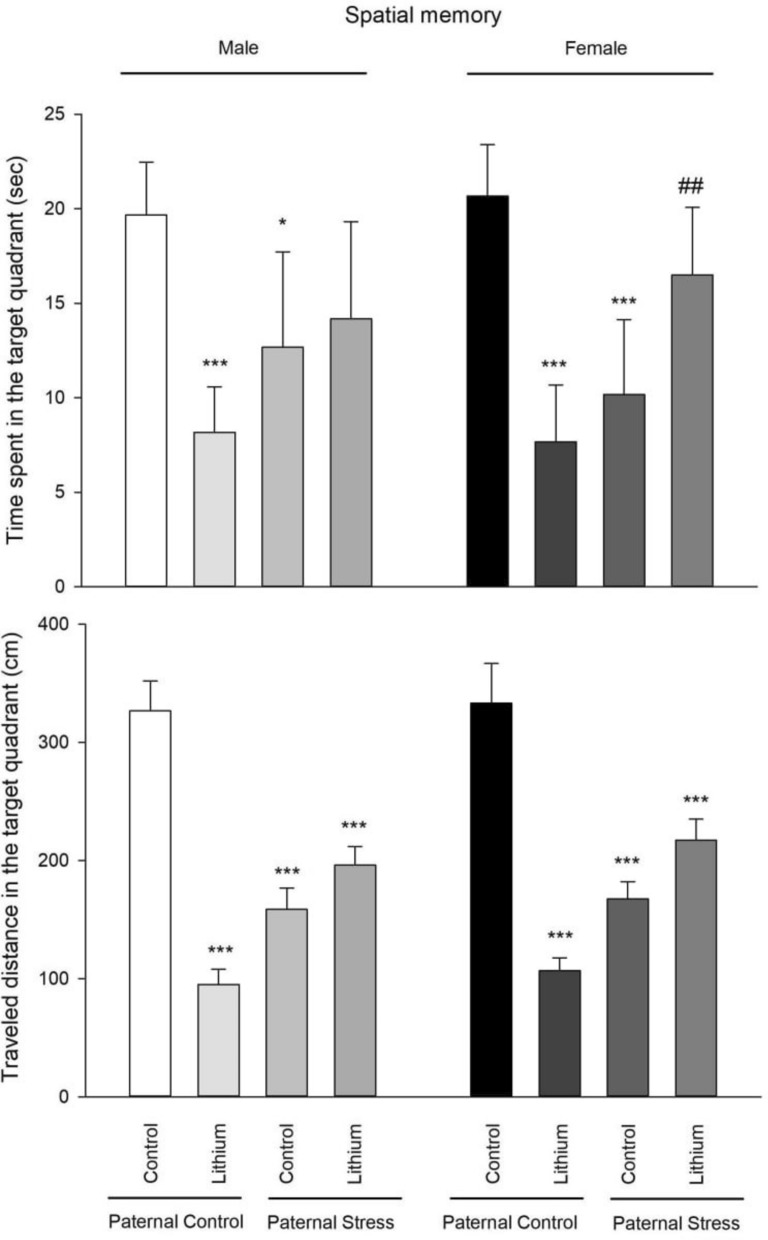
This figure illustrates the results from the Morris water maze, gauging spatial memory across all tested groups (n = 6). Rats were given lithium intraperitoneally at a dose of 50 mg/kg from 21–41 PND, while control rats were administered saline. Noteworthy statistical differences include: ***P < 0.001 and *P < 0.05 when compared to its’ related control group of paternal control; and ##P < 0.01 when compared to respective male group

Post hoc Tukey test also showed that lithium and paternal PTSD decreased time spent in the target quadrant in control rats (P = 0.000) in comparison with control rats. Lithium increased time spent in the target quadrant in paternal PTSD rats in comparison with paternal PTSD rats (P = 0.009). Also, there were no differences between males and females (Fig. 7).

Spatial memory (traveled distance)- The results of one-way ANOVA showed that there was a significant difference between male groups (F3,23 = 166.71, P = 0.000). Post hoc Tukey test also showed that traveled distance in the target quadrant was decreased in all groups (P = 0.000) in comparison with control rats. The results of one-way ANOVA showed that there was a significant difference between female groups (F3, 23 = 123.57, P = 0.000). Post hoc Tukey test also showed that traveled distance in the target quadrant was decreased in all groups (P = 0.000) in comparison with control rats. Also, there were no differences between males and females (Fig. 7).

### BDNF

**Fig. 8:**
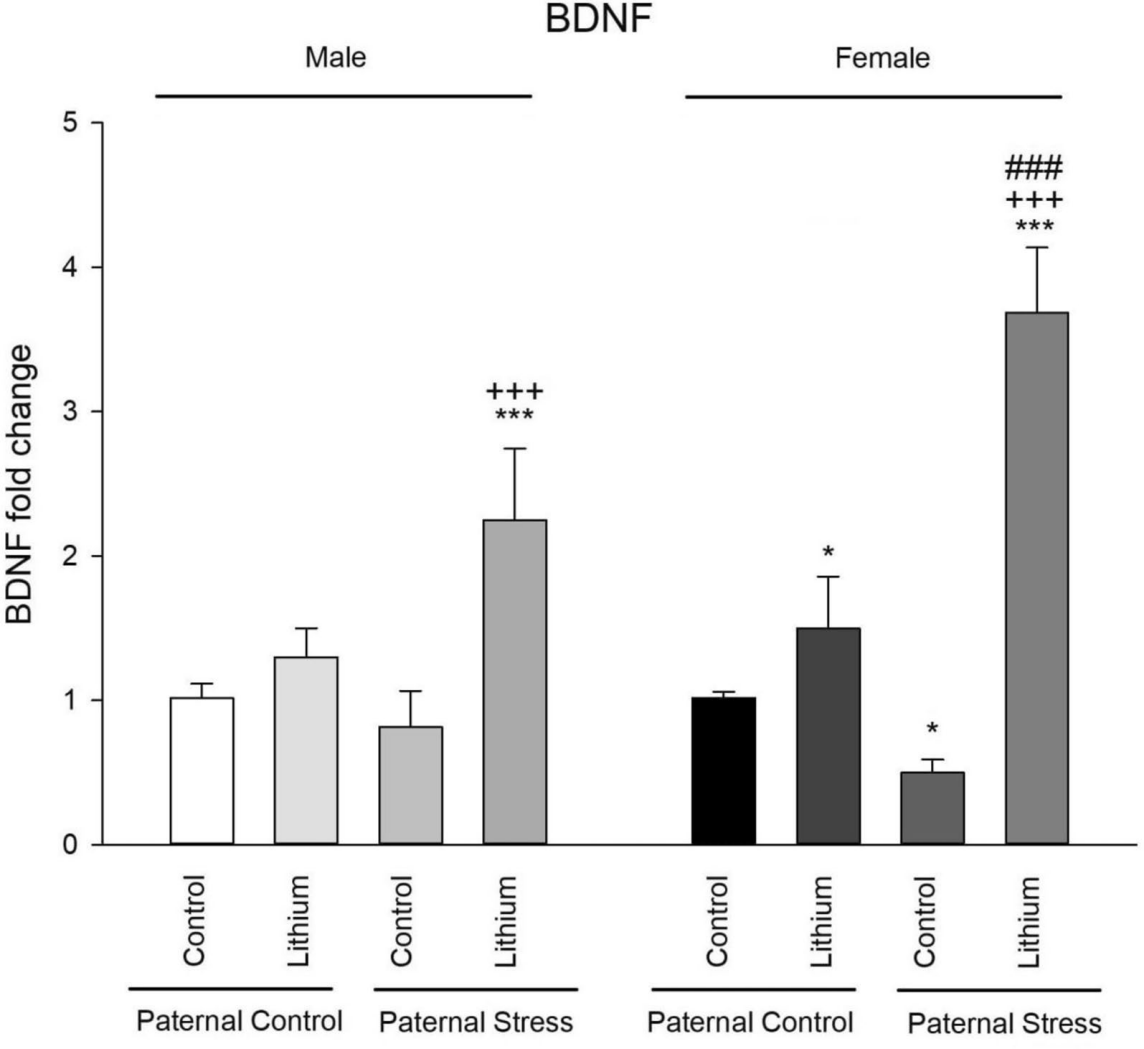
Presented here are the outcomes from the real-time PCR, which measures BDND expression levels in the hippocampus for all groups (n = 6). Rats received an intraperitoneal injection of lithium at 50 mg/kg from 21 to 41 PND. Control rats, on the other hand, were given a saline solution. Significant markers are: ***P < 0.001 and *P < 0.05 when compared to its’ related control group of paternal control; ###P < 0.001 when compared to respective male group; and +++P < 0.001 when compared to respective male group

The results of one-way ANOVA showed that there was a significant difference between male groups (F3, 23 = 27.27, P = 0.000). Post hoc Tukey test also showed that lithium increased BDNF hippocampal expression in paternal PTSD rats (P = 0.000) in comparison with control group of paternal or control father. The results of one-way ANOVA showed that there was a significant difference between female groups (F3, 23 = 136.96, P = 0.000). Post hoc Tukey test also showed that lithium increased BDNF hippocampal expression in paternal control rats (P = 0.044) in comparison with control rats. Also, BDNF expression level was decreased in paternal PTSD rats (P = 0.029) in comparison with control rats. In addition, lithium reversed decreased BDNF level in paternal PTSD rats (P = 0.000). Furthermore, independent t-test showed increased BDNF level in female paternal PTSD rats more than males (P = 0.000). There were no other differences between males and females (Fig. 8).

## Discussion

In the present study, paternal stress induced by a PTSD-like footshock protocol resulted in pronounced behavioral and cognitive alterations in adolescent offspring, with females showing greater vulnerability. Specifically, paternal stress reduced locomotor activity, increased anxiety-like behavior, impaired pain sensitivity, and disrupted learning and memory performance, suggesting long-lasting transgenerational consequences of preconception stress exposure. Lithium treatment partially attenuated these behavioral deficits, particularly in stress-exposed female offspring, indicating a sex-dependent therapeutic responsiveness. Importantly, these behavioral alterations were accompanied by reduced hippocampal BDNF expression in female offspring, suggesting that impaired neurotrophic support may underlie the observed phenotypes. Consistent with previous evidence, these findings highlight the sensitivity of adolescent brain development to paternal stress history and emphasize the modulatory role of lithium on stress-induced neurobehavioral dysfunctions.

Note on exposure window. While much of the literature has focused on maternal prenatal stress, the present study specifically examined paternal preconception stress (i.e., stress exposure of sires before mating), with no contact between sires and dams during gestation or with pups during the pre-weaning phase [43].

Stressful or trauma experiences, particularly in the prenatal period, may increase the development of different neuropsychiatric diseases such as depression and anxiety disorders during adolescence [55]. Importantly, brain development is extremely sensitive to external adverse events and trauma during the prenatal period [56]. It has been shown that prenatal stress is related to a wide range of brain and behavioral alterations in offspring that may persist into adulthood [22]. In this regard, there is large evidence showing the deleterious effects of prenatal stress on cognitive and behavioral functions of the offspring. For example, clinical and preclinical research has shown that chronic stress or trauma during gestation significantly decreases brain gray matter and increases stress hormones during and beyond pregnancy, leading to neurodevelopmental and neuropsychiatric disorders in the offspring [57–59]. Trauma or stressful experiences during pregnancy enhance the level of stress-related hormones in the mother and fetus [60, 61]. Prenatal stress increases the susceptibility to childhood and adult psychopathologies related to attention, learning, depressive symptoms, and brain structural changes in the offspring [60, 62]. A previous study has shown that prenatal stress greatly increases vulnerability to depression in adult rats [63]. It has also been revealed that adult animals exposed to prenatal stress show depression-like or anxiety-like behaviors along with HPA axis dysfunction and impaired neural plasticity [64, 65]. Furthermore, maternal stress in rats leads to spatial memory decline, reduced medial prefrontal cortex spine density, and decreased hippocampal volume in male (but not female) offspring [66]. Maternal restraint stress during gestation also leads to depressive-like behavior in offspring [23]. In addition, paternal preconception stress alters ethanol drinking behavior in male mouse offspring [67].

BDNF, one of the most important neurotrophins in the CNS, plays a key role in synaptic plasticity, cognition, and mood regulation [68, 69], and it is significantly affected by prenatal stress [70]. However, findings are somewhat controversial. Maternal separation, for instance, increases BDNF levels in adolescent rats as a potential protective mechanism against cognitive decline [71, 72]. On the other hand, prenatal stress decreases BDNF expression in the hippocampus and amygdala in rats [73], and significantly reduces BDNF expression while attenuating social behavior in periadolescent rats [74]. It has also been shown that prenatal stress reduces BDNF protein levels in the hippocampus at postnatal day 5, but not at days 8 and 15, and that BDNF protein levels may even increase in adult male rats that experienced prenatal stress [75]. Notably, it has been suggested that different processing of BDNF after prenatal stress in different rat strains has implications for humans, where genetic differences may protect or worsen the impact of prenatal stress [76]. Our data showed that paternal preconception stress decreased BDNF expression in the hippocampus of female offspring; however, lithium significantly increased BDNF expression in both male and female offspring of PTSD-exposed fathers.

Lithium is one of the oldest drugs prescribed for treating psychiatric disorders. Lithium is frequently used to treat bipolar disorder in adults [77]. Furthermore, lithium has been considered both a mood stabilizer and an antidepressant [78]. Of note, it has been reported that lithium administration to a normal brain can have lasting deleterious effects [79]. Lithium also affects cognitive functions and mood state [80]. It has been shown that lithium reverses depressive-like behaviors and augments the effect of selective serotonin reuptake inhibitors (SSRIs) in treatment-resistant depressed rats [81]. In the context of our findings, where lithium was able to alleviate certain depressive-like behaviors in the offspring, it aligns with this broader narrative. The ameliorative effects of lithium in our PTSD paternal model—especially regarding depressive symptoms—resonate with observations of its role in reversing depressive behaviors in rats [81]. While our findings highlight the potential of lithium in modulating anxiety-like behaviors in the offspring, the effects in our study were relatively modest, particularly in the paternal PTSD groups. Our study showed that OCD-like behaviors remained largely unchanged by either paternal stress or lithium treatment. To our knowledge, existing literature does not address lithium’s effects on obsessive–compulsive behaviors; thus, our findings offer a preliminary insight and highlight the need for further research in this direction.

Lithium may reverse the impairing effect of sleep deprivation on social interaction memory in rats [80]. It has been reported that chronic lithium treatment can enhance learning and memory performance in a rat model of Alzheimer’s disease [82]. On the other hand, lithium can affect BDNF function and level. Previous research has shown that lithium significantly decreases infarction volume in the ischemic brain and improves behavioral functions 28 days post-insult, accompanied by increased BDNF levels in cultured cortical neurons [83]. Additionally, lithium treatment upregulates hippocampal claudin-5 and BDNF protein expression in stressed rats [84]. Chronic lithium treatment has also been shown to increase BDNF levels in the hippocampus of obese rats [85]. Other studies have demonstrated that lithium induces neuroprotective and pro-cognitive effects by suppressing glycogen synthase kinase-3 (GSK-3), increasing BDNF levels, and reducing pro-apoptotic factors [86, 87]. The results of the present study also showed that lithium significantly increased BDNF levels in the hippocampus of male and female offspring with a history of paternal chronic footshock exposure. Lithium likewise increased BDNF levels in the hippocampus of control female offspring. Moreover, lithium significantly reversed most of the impaired cognitive and behavioral functions in male and female offspring with a history of paternal chronic footshock exposure. Lithium has been suggested to alleviate irritability in PTSD patients and even to prevent PTSD development post-trauma [30, 88]. While studies emphasize lithium’s positive impact, its diminished efficacy in PTSD–bipolar comorbidity presents challenges [32]. Our research corroborates lithium’s therapeutic promise, showing its capacity to counter cognitive-behavioral adversities in offspring due to a paternal PTSD-like model. However, the nuanced gender-specific effects—especially in female offspring—underscore the complexity of lithium’s role. Thus, while both prior studies and our results laud lithium’s potential, they simultaneously highlight the need for a discerning approach to its application. Notably, lithium did not uniformly reverse all behavioral impairments, and its effects were dependent on both sex and behavioral domain, indicating that lithium’s modulatory influence may be limited to specific neural circuits and stress-sensitive phenotypes [88, 89].

## Conclusion

In conclusion, paternal PTSD-like stress induces persistent cognitive and behavioral impairments in adolescent offspring, with females displaying heightened vulnerability. These alterations are associated with decreased hippocampal BDNF expression, suggesting impaired neuroplastic mechanisms. Postnatal lithium treatment partially mitigates these effects in a sex-dependent manner, highlighting its potential therapeutic relevance. Collectively, these findings underscore the importance of paternal mental health prior to conception and provide novel evidence for lithium’s role in modulating transgenerational stress effects.

## Data Availability

The datasets generated and analyzed during this study are available from the corresponding author upon reasonable request.
